# Serial crystallography on *in vivo* grown microcrystals using synchrotron radiation

**DOI:** 10.1107/S2052252513033939

**Published:** 2014-02-10

**Authors:** Cornelius Gati, Gleb Bourenkov, Marco Klinge, Dirk Rehders, Francesco Stellato, Dominik Oberthür, Oleksandr Yefanov, Benjamin P. Sommer, Stefan Mogk, Michael Duszenko, Christian Betzel, Thomas R. Schneider, Henry N. Chapman, Lars Redecke

**Affiliations:** a Center for Free-Electron Laser Science (CFEL), Deutsches Elektronensynchrotron (DESY), Notkestrasse 85, 22607 Hamburg, Germany; b European Molecular Biology Laboratory (EMBL), Hamburg Outstation, Notkestrasse 85, 22607 Hamburg, Germany; c Joint Laboratory for Structural Biology of Infection and Inflammation, Institute of Biochemistry and Molecular Biology, University of Hamburg, and Institute of Biochemistry, University of Lübeck, Notkestrasse 85, 22607 Hamburg, Germany; d Institute of Biochemistry and Molecular Biology, University of Hamburg, Notkestrasse 85, 22607 Hamburg, Germany; e Interfaculty Institute of Biochemistry, University of Tübingen, Hoppe-Seyler-Strasse 4, 72076 Tübingen, Germany; f Institute of Experimental Physics, University of Hamburg, Luruper Chaussee 149, 22761 Hamburg, Germany

**Keywords:** protein microcrystallography, serial crystallography, *in vivo* grown microcrystals

## Abstract

Crystal structure determinations of biological macromolecules are limited by the availability of sufficiently sized crystals and by the fact that crystal quality deteriorates during data collection owing to radiation damage. Exploiting a micrometre-sized X-ray beam, high-precision diffractometry and shutterless data acquisition with a pixel-array detector, a strategy for collecting data from many micrometre-sized crystals presented to an X-ray beam in a vitrified suspension is demonstrated. By combining diffraction data from 80 *Trypanosoma brucei* procathepsin B crystals with an average volume of 9 µm^3^, a complete data set to 3.0 Å resolution has been assembled. The data allowed the refinement of a structural model that is consistent with that previously obtained using free-electron laser radiation, providing mutual validation. Further improvements of the serial synchrotron crystallography technique and its combination with serial femtosecond crystallography are discussed that may allow the determination of high-resolution structures of micrometre-sized crystals.

## Abstract 2

The structure solution of *T. brucei* cathepsin B from 80 *in vivo* grown crystals with an average volume of 9 µm^3^ obtained by serial synchrotron crystallography at a microfocus beamline is reported.

## 1. Introduction   

Macromolecular crystallography (MX) is a powerful method for obtaining structural information about biological macromolecules and their assemblies. Since the 1990s, advanced third-generation synchrotrons have been used to produce micrometre-sized high-flux X-ray beams whose focus size matches the size of small crystals (Cusack *et al.*, 1998 ▶; Riekel *et al.*, 2005 ▶; Evans *et al.*, 2011 ▶; Smith *et al.*, 2012 ▶). X-ray beams with dimensions of less than 10 µm are now in routine use at many synchrotron-radiation facilities (Evans *et al.*, 2011 ▶) and enable the determination of crystal structures from crystals with volumes of less than 1000 µm^3^ (Cusack *et al.*, 1998 ▶). Using these microbeams, the structures of the cypovirus polyhedra protein (Coulibaly *et al.*, 2007 ▶), amyloid-like fibres (Nelson *et al.*, 2005 ▶) and a number of complexes addressing the structure and function of G-protein-coupled receptors (GPCRs; Cherezov *et al.*, 2007 ▶; Rasmussen *et al.*, 2007 ▶, 2011 ▶) have been determined from micrometre-sized crystals.

Inelastic and absorption events deposit energy in the crystal, triggering a variety of chemical reactions that lead to a repositioning of atoms (during the experiment), ultimately reducing the crystalline order and corresponding to increasing levels of ‘radiation damage’. As a consequence, even under cryogenic conditions, the quality of the diffraction data, in particular those at high resolution, quickly deteriorates as a function of the X-ray dose applied to the crystal. Since the earliest days of macromolecular crystallography, this problem has been addressed by combining partial data sets collected from multiple crystals into a full data set to high resolution. In the 1990s cryogenic cooling became routine in macromolecular crystallography (Henderson, 1990 ▶; Garman & Schneider, 1997 ▶), extending the lifetime of individual crystals, but with the implementation of undulator beamlines on third-generation synchrotrons the tolerable X-ray dose could be deposited in a short time even for cryocooled crystals (Ravelli & Garman, 2006 ▶). Data-collection schemes employing multiple positions on a single crystal (Riekel *et al.*, 2005 ▶), to distribute the delivered dose over the entire crystal, or partial data sets from multiple crystals (Brodersen *et al.*, 2003 ▶) were developed. The main complication with multicrystal methods is that systematic differences between data measured on different crystals, or at different positions on a single crystal, can prevent the derivation of a consistent assembled data set. Nevertheless, Hendrickson and coworkers (Liu *et al.*, 2012 ▶) have recently shown that by removing sources of experimental error and by carefully selecting consistent partial data sets for merging, data sets of sufficient accuracy for phasing using the anomalous signal from S atoms in native proteins can in fact be assembled. Systematic errors in data collections from multiple positions in the same crystal can, amongst other techniques, be reduced by ‘helical scan’ procedures (Flot *et al.*, 2010 ▶), in which a single crystal, usually needle-shaped, is continuously moved during data collection to expose fresh parts of the same crystal.

When working with small crystals, it is often not possible to locate the crystals accurately using optical microscopy owing to their small size, optical distortions caused by materials surrounding the crystal, or the crystal being embedded in an opaque matrix. All of these problems are present during the data collection of GPCR crystals grown from lipidic cubic phase (LCP). Cherezov *et al.* (2009 ▶) described a method in which the mounted LCP containing crystals is first systematically rastered using a small beam to test each position for diffraction. Data are then collected in a second step using the rotation method (Arndt & Wonacott, 1977 ▶) from positions where diffraction was actually detected. The application of this method has been crucial in the structure determination of GPCRs, where the crystals are small (of the order of 25 × 6 × 4 µm^3^) and invisible to optical light because the lipidic mesophase in which the crystals grow turns opaque upon cryogenic cooling. Recently, an alternative procedure was proposed in which radiographs are taken of the entire sample mount to localize crystals (Warren *et al.*, 2013 ▶).

Extremely short pulses of X-rays, each of tens of femto­seconds duration or even shorter, generated by a free-electron laser (FEL), have recently been shown to overcome the dose limitations mentioned above. Referred to as ‘diffraction before destruction’, the inertia of the atoms prevents significant atomic displacement during the short exposing pulse (Neutze *et al.*, 2000 ▶; Boutet *et al.*, 2012 ▶; Barty *et al.*, 2012 ▶), even though the initial photoabsorption eventually leads to plasma formation and the complete vaporization of the sample. A typical serial femtosecond crystallography (SFX) experiment collects tens or hundreds of thousands of single-crystal X-ray diffraction ‘snapshots’ from a liquid suspension of protein microcrystals that flows across the focused X-ray beam at room temperature (Chapman *et al.*, 2011 ▶). Since each FEL pulse destroys the sample, only one diffraction pattern can be collected per crystal. New data-processing tools, such as *Cheetah*, *CrystFEL* (White *et al.*, 2012 ▶, 2013 ▶; Kirian *et al.*, 2011 ▶) and *cctbx.xfel* (Kern *et al.*, 2013 ▶), have been developed to process these large data volumes and have been successfully applied to several examples (Chapman *et al.*, 2011 ▶; Boutet *et al.*, 2012 ▶; Redecke *et al.*, 2013 ▶; Kern *et al.*, 2013 ▶).

The crystal structure of *Trypanosoma brucei* procathepsin B (TbCatB) in complex with its native propeptide represents the first novel bioinformation obtained by applying the SFX approach at a free-electron laser (Redecke *et al.*, 2013 ▶). This enzyme is of scientific and medical interest, since the knockout of its encoding gene has been shown to be lethal to the parasite that causes sleeping sickness in Africa (Abdullah *et al.*, 2008 ▶; Bryant *et al.*, 2009 ▶), which turns TbCatB into an urgently required potential new drug target (Fairlamb, 2003 ▶). TbCatB crystals grew spontaneously within living baculovirus-infected insect cells during protein overexpression to a size of 10–15 µm in the longest dimension (Koopmann *et al.*, 2012 ▶). In a recent experiment (Redecke *et al.*, 2013 ▶), these crystals were used to obtain 178 875 single-crystal diffraction patterns by SFX at LCLS, which enabled structure determination of the enzyme to 2.1 Å resolution.

Inspired by the SFX methodology and the new capabilities to process large data sets, as well as the successful structure determination of TbCatB in the pro-form using the SFX approach, we embarked on an experiment to determine the crystal structure of *T. brucei* procathepsin B using a suspension of *in vivo* grown microcrystals mounted in a standard nylon loop for crystallographic data collection on a microfocus synchrotron beamline. Owing to the presence of cell remnants in the *in vivo* crystal suspension, individual TbCatB crystals are difficult to detect in the cryocooled sample. Our procedure combined elements from SFX with a helical line-scan approach used in microcrystallography. As with the SFX approach, an initially unknown subset of the recorded detector frames contain diffraction signals, which are selected for further processing into a crystallographic data set. Unlike the snapshots recorded at an FEL, the sample is rotated during exposure and multiple exposures from the same crystal can be obtained and subsequently processed in a consistent manner. In the following, we describe the diffraction experiment, data processing and structure determination. The independent determination of the crystal structure of pro-cathepsin B from *T. brucei via* two different methods using synchrotron and FEL radiation sources provided a unique opportunity to validate the results obtained against each other, and we present a comparison of the crystallographic models.

## 2. Results   

### 2.1. Sample preparation and data collection   

**Figure 1:**
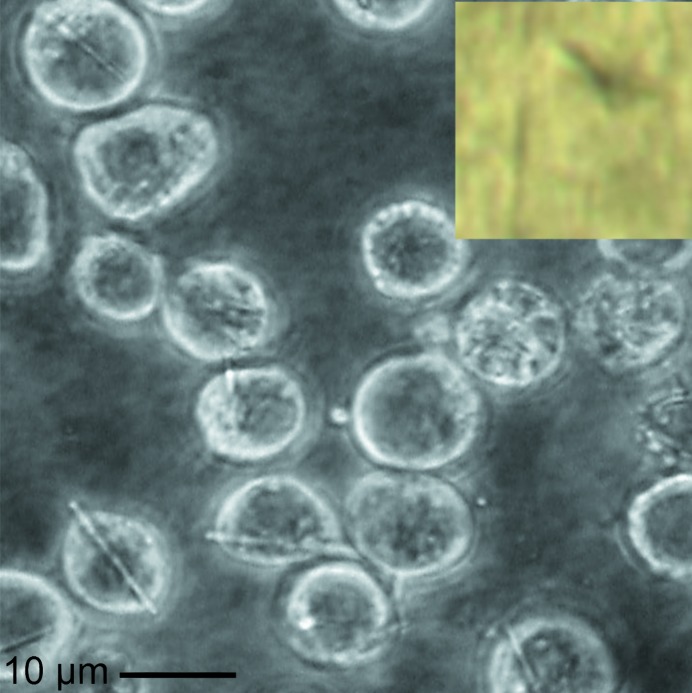
Light micrograph of Sf9 cells spontaneously crystallizing trypanosomal cathepsin B. The isolated and purified crystals (inset) were mounted on a standard cryoloop for the serial synchrotron diffraction experiments.

Spontaneous crystallization of TbCatB was obtained in baculovirus-infected Sf9 insect cells following our previously established protocol (Fig. 1 ▶; Koopmann *et al.*, 2012 ▶). The *in vivo* crystals were isolated and purified by cell lysis and stepwise centrifugation based on their high mechanical and chemical stability. The purity of the crystal preparation was verified by scanning electron microscopy (SEM). Prior to diffraction data collection, the suspension of needle-shaped crystals containing approximately 5 × 10^8^ crystals per millilitre with average dimensions of 0.9 × 0.9 × 11 µm (approximately 9 µm^3^ in volume) was supplemented with 40%(*v*/*v*) glycerol as a cryoprotectant. A small volume of approximately 13 nl of the crystalline suspension was mounted after settling in a standard 20 µm thick nylon loop (Hampton Research, USA; 0.7 mm diameter) containing approximately 5000 crystals.

Diffraction experiments were conducted on the P14 microfocus beamline at the PETRA III storage ring (DESY, Hamburg) with a 4 × 5 µm (FWHM) microfocus beam, a total photon flux of 1.2 × 10^12^ photons s^−1^ at the sample position and a photon energy of 10.00 keV. The nylon loop was mounted on an MK3 mini-kappa goniometer head attached to an MD3 microdiffractometer (ARINAX, Moirans, France) and kept at 110 K using a gaseous nitrogen stream (Cryojet XL, Oxford Instruments, England). Diffraction data were recorded on a PILATUS 6M-F detector (DECTRIS Ltd, Baden, Switzerland).

In a set of initial experiments, the diffraction properties of individual TbCatB crystals were characterized. After optically centring selected crystals with respect to the X-ray beam, single diffraction images collected with an oscillation range of 1° at an exposure time of 2 s showed diffraction spots extending to a resolution higher than 3 Å (Supporting Fig. S2). Using a series of short rotation exposures, by visual inspection of diffraction images and using the ability to index them as a criterion (see Supporting Information), we empirically determined a crystal lifetime of 0.5–1 s under the conditions at hand. This observed crystal lifetime corresponded well to the lifetime of 0.88 s calculated *via* *RADDOSE* (Paithankar & Garman, 2010 ▶) at an estimated maximum dose rate of 34 MGy s^−1^.

**Figure 2:**
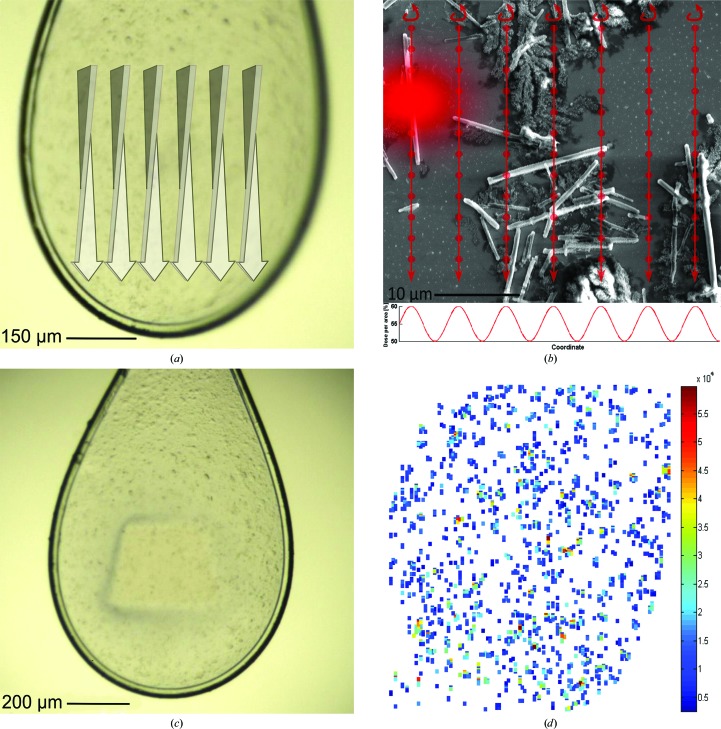
Experimental setup of the serial synchrotron crystallography experiment. (*a*) Schematic macroscopic illustration of the serial helical line-scan approach using a standard cryogenic loop, imaged with the inline microscope. (*b*) SEM image of isolated *in vivo* grown cathepsin B microcrystals on a silicon support. Red arrows illustrate the serial helical line scan. The incident beam is represented by the red ‘flare’. The colour density in the flare is proportional to a calculated two-dimensional Gaussian function with FWHM 4 × 5 µm, with relative size to the 10 µm scale bar, showing a significant fraction of photon flux away from the centre of the beam. Red dots illustrate the positions of collected frames during the line scan with an oscillation width of 0.5° each. The graph (lower part) visualizes the delivered dose per area against arbitrary coordinates, indicating a total dose per area fluctuating between 50 and 60% owing to the ratio of FWHM of the beam and the gap between each line-scan position. (*c*) After the serial helical line scan, the photoinduced ionization at the exposed part of the sample is macroscopically visible. (*d*) Heatmap of diffraction images in the crystal loop after pre-selection using *CrystFEL*. The colour bar codes the average intensity of Bragg peaks in each diffraction pattern as an indication of the diffraction strength in each pattern.

To collect a complete data set, we employed a data-collection strategy in which a region of interest of 600 × 600 µm was raster scanned with rotation exposures (Fig. 2 ▶). 120 parallel helical scans were performed, spaced 5 µm apart. During each helical scan, the goniostat was rotated from Ω = −45° to Ω = +45° (where Ω = 0° corresponds to the orientation of the loop surface perpendicular to the incoming beam) and translated by 600 µm. Taking 240 exposures of 1 s duration over the course of a each helical scan, each individual frame recorded on the detector corresponded to a rotation of 0.375° and a translation of 2.5 µm of the sample. Under these conditions, every crystal within the region of interest received a dose of between 50 and 60 MGy. This high total (integrated over multiple exposures) dose was chosen to collect the highest possible resolution data from each crystal. Using the above strategy, 28 800 detector frames were acquired in a period of 8 h.

### 2.2. Data processing and structure determination   

Adopting the recently established methods for the processing of diffraction patterns from SFX experiments, the *CrystFEL* software suite (White *et al.*, 2012 ▶) was used as a first step to identify and index single-crystal diffraction patterns within the large set of detector frames. Frames containing diffraction patterns that were recorded consecutively during the same helical scan were considered as originating from the same crystals and were assembled into 595 groups containing between two and ten consecutive frames (Fig. 2 ▶ *d* and Supporting Fig. S2). These groups were further treated as regular rotation data for re-indexing and integration applying *XDS* (Kabsch, 2010 ▶). Horizontally adjacent groups of diffraction images, potentially containing diffraction patterns from the same crystal, were treated independently. In a standard three-dimensional profile-fitting procedure, both fully and partially recorded reflections were integrated. Processing was successful for 130 groups containing a total of 557 frames.

After iterative merging and scaling, 109 661 reflection intensities in the resolution range from 88 to 3.0 Å were merged into a final data set consisting of 8881 merged reflection intensities with an overall completeness of 99.8%. This final set of reflection intensities included data from 426 diffraction patterns collected from 80 individual TbCatB crystals in 120 groups. The distribution of the size of the groups (Supporting Fig. S2) apparently reflects the variation in the crystal size in TbCatB preparations (see Fig. S1 of Redecke *et al.*, 2013 ▶). Most of the data were derived from groups of three to five consecutive frames corresponding to a total rotation range of 1.125–1.875° of one crystal. A small fraction of data originated from groups containing eight to ten frames, while the majority of groups contained three to five consecutive frames.

The quality and internal consistency of the data were judged on the basis of standard 〈*I*/σ(*I*)〉 statistics and on the basis of the CC* criteria recently advocated as a single statistically valid guide for deciding the resolution cutoff of the obtained data (McCoy *et al.*, 2007 ▶; Karplus & Diederichs, 2012 ▶; Evans, 2012 ▶). The CC* calculated in resolution shells for the TbCatB data set (Supporting Fig. S1) indicated the presence of statistically significant data to a resolution of 3.0 Å and below.

Following the same strategy as for the previous determination of the *T. brucei* pro-cathespsin B crystal structure *via* SFX (Redecke *et al.*, 2013 ▶), initial phases were obtained by molecular replacement with *Phaser* (McCoy *et al.*, 2007 ▶) using the structure of the nonglycosylated and *in vitro* crystallized TbCatB (PDB entry 3mor; Koopmann *et al.*, 2012 ▶) that lacks the propeptide and the carbohydrate chains as a search model. During stepwise model building and refinement, 62 propeptide residues and five carbohydrate residues were manually placed in difference electron-density maps.

**Figure 3:**
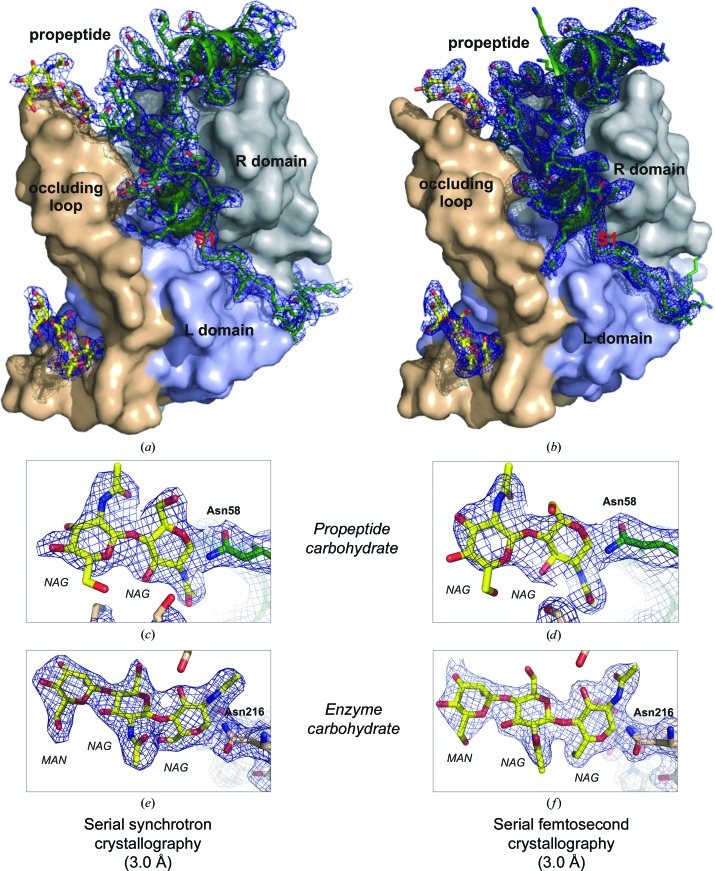
Quality of the calculated electron density from diffraction data sets of *in vivo* grown TbCatB crystals collected using serial synchrotron crystallography (3.0 Å resolution; left) and SFX (refined at 3.0 Å resolution; PDB entry 4hwy; right) techniques. (*a*, *b*) Surface representation of the TbCatB–propeptide complexes independently solved by molecular replacement using the mature TbCatB structure (Koopmann *et al.*, 2012 ▶) as a search model. The solutions consistently revealed additional electron density (2*F*_obs_ − *F*_calc_, 1σ, blue) of the propeptide (green) that is bound to the V-shaped substrate-binding cleft and of two carbohydrate structures (yellow) N-linked to the propeptide (*c*, *d*) and to the mature enzyme (*e*, *f*). Considering the difference in maximum resolution, the propeptide, as well as both carbohydrates, are well defined within the electron-density maps, confirming that the phases are not biased by the search model.

The refined TbCatB structure (*R* factor = 22.3%, *R*_free_ = 26.4%) shares the papain-like fold which is characteristic of cathepsin B enzymes, including the propeptide residues 27–72 and 79–85 without defined electron density in between, as well as a carbohydrate chain consisting of two *N*-acetylglucosamine (NAG) monomers N-linked to Asn58 (in the propeptide) and another carbohydrate chain consisting of two NAG monomers and one β-­mannose (BMA) molecule N-linked to Asn216 of the enzyme domain. Overall, the 3.0 Å resolution electron-density map is well defined by the TbCatB model. No electron density is observed for nine flexible amino-acid side chains mainly located within a loop region spanning residues His195–Asn209 or for ten atoms of the carbohydrate structures. In particular, as for the SFX structure determination, the expected features of the electron-density map that were not part of the search model are well defined by the propeptide and two carbo­hydrate chains after manual model building and refinement (Fig. 3 ▶).

### 2.3. Comparison of the structural TbCatB models   

For detailed comparison of the *T. brucei* procathepsin B structure solved in this study at 110 K using synchrotron radiation with that previously obtained at room temperature using the FEL-based SFX technique (PDB entry 4hwy; Redecke *et al.*, 2013 ▶), electron-density maps were generated using the SFX data truncated at 3.0 Å resolution. Applying an identical refinement protocol that omits solvent atoms resulted in an *R* factor of 17.0% (*R*_free_ = 19.6%). A slight shrinking of the unit-cell parameters of the TbCatB *in vivo* crystals observed for the synchrotron data set (Table 1 ▶) can be attributed to the cryogenic data-collection conditions. At room temperature, unit-cell parameters of *a* = *b *= 125.5, *c* = 54.6 Å were previously obtained by SFX. The superposition of the peptide backbone atoms of both structures revealed a high degree of consistency, resulting in an average r.m.s.d. value of 0.35 ± 0.19 Å, which is comparable to the overall coordinate error of 0.32 Å estimated based on maximum likelihood by *REFMAC*5.5 (Murshudov *et al.*, 2011 ▶). No significant structural differences are present, including no major features related to radiation damage (Supporting Fig. S3). Main-chain deviations of more than 0.8 Å are limited to nine residues located at the N-terminus and C-­terminus, in flexible loop regions and at positions flanking the disordered part of the propeptide region that results from an increased flexibility of the residues after proteolytic cleavage between Ser78 and Ile79 (Redecke *et al.*, 2013 ▶). Even the two carbohydrate chains are clearly defined and largely superimposable between the two models (Figs. 3 ▶ *c*–3 ▶ *f*). Slight differences were only observed for the second *N*-­acetyl­glucosamine residue of the propeptide carbohydrate, which represents the most flexible carbohydrate within the model. This is further reflected by the almost identical number of amino-acid side chains/carbohydrate atoms not defined by electron density in both TbCatB structures (nine side chains and ten carbohydrate atoms in this structure *versus* 11 side chains and eight carbohydrate atoms in the SFX structure).

Despite the overall similarity in atomic coordinates, systematic differences were observed in the relative heights of the electron-density peaks at the 12 Cys SG atoms involved in disulfide bridges. Considering refined Debye–Waller factors as an (anticorrelated) measure of the height of electron-density maxima, we note that in the synchrotron structure the average Debye–Waller factor (〈*B*_SG_〉 = 57 Å^2^) is higher than that averaged over all atoms (〈*B*_All_〉 = 41 Å^2^). In the SFX TbCatB structure refined using an identical protocol at 3.0 Å resolution, 〈*B*_SG_〉 (38 Å^2^) is lower than 〈*B*_All_〉 (45 Å^2^). This observation is consistent with a significant diffraction contribution from reduced disulfide bonds in the synchrotron data but not in the SFX data.

## 3. Discussions and conclusions   

Particularly for crystals with dimensions in the low micometre range, the determination of macromolecular crystal structures is inherently limited by radiation damage. In most cases, when an X-ray flux sufficient to measure Bragg reflections to the highest resolution as defined by the degree of crystalline order in a given crystal is used, the crystal will be severely damaged before complete diffraction data can be collected. As a consequence, in practice a compromise is sought balancing the resolution and the completeness of the data to be measured on a single crystal. In recent years, combining data from multiple crystals has enabled the determination of a number of important structures (Rasmussen *et al.*, 2011 ▶; Siu *et al.*, 2013 ▶; Li *et al.*, 2013 ▶), despite the difficulties arising from systematic errors when data from multiple crystals are merged. The recent introduction of SFX exploiting X-rays from an FEL to collect single diffraction images to the maximum resolution from large numbers of crystals in the ‘diffraction-before-destruction’ regime has realised an extreme approach to overcoming the radiation-damage problem.

Here, we have demonstrated a strategy for the collection of complete diffraction data close to the diffraction limit from micrometre-sized crystals using synchrotron radiation. This method is based on the serial illumination of subvolumes of a sample consisting of a cryogenically vitrified suspension of microcrystals mounted in a standard nylon loop. During exposure, the sample is rotated, resulting in ‘classical’ rotation frames (Arndt & Wonacott, 1977 ▶) for subvolumes presenting crystalline material to the X-ray beam. The X-ray dose received by an exposed subvolume is chosen to fully exploit the crystal lifetime during the exposure time used, a requirement to achieve maximum resolution under these conditions. The combination of microcrystals assuming quasi-random orientations in the suspension and continuous rotation of the loop during each helical scan will present a variety of different crystal orientations to the beam, effectively covering the rotation space and thus providing complete diffraction data in the end.

The crystals used in this study are smaller in volume (∼10^7^ unit cells in a crystal of volume 9 µm^3^) than those of cypovirus polyhedra (CPV) studied by Metcalf and coworkers (Coulibaly *et al.*, 2007 ▶; ∼10^8^ unit cells in 125 µm^3^). In addition, the TbCatB crystals are embedded in a matrix giving rise to a high scattering background, similar to the situation of GPCR crystals mounted in LCP, where the crystals are commonly of larger volume (∼10^9^ unit cells in 600 µm^3^; Cherezov *et al.*, 2007 ▶). While systematic strategies collecting diffraction data on individually pre-centred crystals were employed for both the CPV and GPCR cases, the serial strategy suggested here has delivered data of comparable quality in terms of signal to noise of the measured diffraction intensities without the need to identify and centre micrometre-sized crystals before data collection (see Supporting Information).

The serial synchrotron-radiation diffraction data allowed us to phase and refine the TbCatB structure using standard technologies. With respect to the data set collected from TbCatB using FEL radiation, the data collected at the synchrotron extend to lower resolution, reflecting the difference between measuring radiation-damage-free ‘infinitesimally still’ data with femtosecond laser pulses and a macroscopic rotation of the crystal over 0.375°, with a similar dose in both cases. Additionally, different levels of background influence the data quality. While a liquid jet of approximately 4 µm diameter delivered the sample for SFX, a 20 µm nylon loop suspending a film of 20 µm thickness was used to mount the crystals in this study. The structural model built against the synchrotron data is consistent with the model obtained with FEL radiation, providing mutual validation.

The present implementation of the serial synchrotron strategy can be improved in many ways. The embedding of the TbCatB crystals with a maximum dimension of approximately 10 µm in a vitrified matrix of approximately 20 µm thickness consisting of buffer and cell debris causes significant background scatter that could be minimized by employing different mounting technologies, such as adaptations of cryo-EM techniques (Nederlof *et al.*, 2013 ▶) or graphene supports (Wierman *et al.*, 2013 ▶). Reducing the beam size to 1 × 1 µm will reduce the background scattering 20-fold. Increasing the flux density of the X-ray beam will reduce the data-collection time, which is currently several hours for a single loop-mounted drop of crystalline suspension. This can be accomplished using improved X-ray optics and the implementation of wide-bandpass monochromators. Merging data collected on multiple loops would increase the signal-to-noise ratio, while decreasing the rotation increment per X-ray dose unit could increase the resolution to which diffraction data can be recorded closer to the limit posed by the degree of order in the crystal.

The method presented here is conceptually simple and could be implemented at many microfocus synchrotron-radiation beamlines employing existing helical scan schemes. It lends itself to data collection on small crystals in suspension, such as those obtained from *in vivo* preparations, as it avoids the centring of hardly visible (or invisible) crystals. Parameters can be tuned to maximize accuracy (*e.g.* applying a larger rotation range per exposed subvolume may allow more accurate integration and scaling) or to maximize resolution (by using a smaller rotation range during the application of the tolerable X-ray dose).

In addition to the promising application as a standalone approach, the combination of serial synchrotron and SFX data collected for a given crystallized protein further offers a new strategy for scaling and phasing of SFX data. In comparison to SFX data collection, serial synchrotron crystallography allows the extraction of accurate diffraction data, albeit to lower resolution owing to the finite rotation range and the onset of radiation damage during the exposure, from a small number of microcrystals by the systematic acquisition of structure-factor amplitudes followed by the application of well defined scaling models modelling a finely controlled experimental process. In contrast, at present, a three orders of magnitude larger number of microcrystals is required for the convergence of Monte Carlo intensity integration when arithmetic means of partially recorded intensities are used without scaling (Kirian *et al.*, 2011 ▶). The use of complete and accurate low-resolution data sets obtained using synchrotron radiation for bootstrapping scaling procedures for SFX data could improve the convergence behaviour of these procedures. If diffraction data can be collected on the same system using X-rays from both synchrotron and free-electron laser sources, the combined use of these data therefore has the potential to provide more accurate crystallographic data than those originating from only one of the two methods, ultimately resulting in higher quality macromolecular structures from micrometre-sized crystals.

## Supplementary Material

PDB reference: cathepsin B, 4n4z

Supporting information. Includes Table S1 as well as Figs. S1, S2 and S3, which provide details of the quality measures of the diffraction data set, an exemplary diffraction pattern, the size distribution in the groups of adjacent indexed frames and details of the structural comparison of the T. brucei procathepsin B structure determined by SFX using free-electron laser radiation and by serial synchrotron crystallography in this study.. DOI: 10.1107/S2052252513033939/jt5002sup1.pdf

## Figures and Tables

**Table 1:** X-ray data-collection and refinement statistics for *in vivo* crystallized TbCatB analyzed at the P14 beamline of the PETRA III synchrotron source (DESY, Hamburg, Germany) Values in parentheses are for the highest resolution shell.

Data collection	
Light source, beamline	PETRA III, P14
Maximum dose (MGy)	50–60
Space group	*P*4_2_2_1_2
Unit-cell parameters (Å)	*a* = *b* = 123.5, *c* = 54.3
*V*_M_ (Å^3^ Da^−1^)	2.99
Solvent content (%)	58.6
Resolution range (Å)	88.1–3.0 (3.16–3.00)
No. of unique reflections	8881
Completeness (%)	99.8 (99.9)
*R*_merge_	0.71 (2.69)
〈*I*/σ(*I*)〉	3.7 (1.0)
CC*	0.97 (0.79)
Multiplicity	12.3 (12.6)
Refinement	
Resolution range (Å)	88.1–3.0
No. of reflections used in refinement	8482
No. of reflections used for *R*_free_	399
*R*_work_/*R*_free_	0.223/0.264
No. of atoms	
Protein	2392
Carbohydrate	67
*B* factors (Å^2^)	
Protein (main chain/side chain)	38/43
Carbohydrate	54
R.m.s. deviations	
Bond lengths (Å)	0.01
Bond angles (°)	1.32
Average r.m.s. *B* factor (main/side chain)	1.6/1.8
Ramachandran plot (%)	
Most favoured	91.2
Allowed	8.2
Disallowed	0.66
