# Supplementary material for: Serial crystallography on in vivo grown microcrystals using synchrotron radiation
Source: IUCrJ. 2014 Feb 10;1(Pt 2):87–94. doi: 10.1107/S2052252513033939 (PMC4062088; doi:10.1107/S2052252513033939)
Supplement: Supplementary file 1 [file m-01-00087-sup1.pdf]

IUCrJ

Volume 1 (2014)

Supporting information for article:

Serial crystallography on *in vivo* grown microcrystals using synchrotron radiation

Cornelius Gati, Gleb Bourenkov, Marco Klinge, Dirk Rehders, Francesco Stellato, Dominik Oberthür, Oleksandr Yefanov, Benjamin P. Sommer, Stefan Mogk, Michael Duszenko, Christian Betzel, Thomas R. Schneider, Henry N. Chapman and Lars Redecke

Supporting Information

Appendix A. Methods

A1. Preparation of TbCatB *in vivo* crystals

TbCatB *in vivo* crystals were grown and purified as previously described (Koopmann et al. 2012), except for the following optimizations: For lysis, the *Sf*9 cells were re-suspended in hypotonic buffer consisting of 10 mM HEPES (pH 7.5), 10 mM sodium chloride and 5 mM magnesium chloride. The suspension was heavily vortexed and supplemented with 25 units mL^{-1} benzonase (Novagen) to reduce viscosity, followed by incubation for 2 hours at 37 °C. For crystal purification, the cell lysate was centrifuged for 10 minutes at 200 xg to separate remaining cell debris and nuclei from the crystals. The micron-sized *in vivo* crystals within the supernatant were pelleted for 15 minutes at 4,500 xg and adjusted to a concentration of 5 x 10^8 crystals mL^{-1} with 1x phosphate buffered saline (PBS).

A2. Data collection

The diffraction experiments on *in vivo* grown TbCatB-crystals in a crystalline suspension were carried out at EMBL beamline P14 on the PETRA III storage ring (DESY, Hamburg) operating at 6 GeV electron beam energy, 80.0 mA current and 1.3 nm*rad horizontal emittance. The U29 undulator was tuned to the maximum brilliance of the first harmonic at 10.00 keV (gap 20.90 mm). This specific choice of photon energy and undulator setting provides a high photon flux at a low power load on the double-crystal monochromator (DCM, Si 111), thus minimizing the variation in beam position and/or beam intensity due to thermal drifts of the DCM during a data collection over the time-course of several hours.

The X-ray beam was focused at the sample position at 61 m from the source using Rh-coated adaptive bimorph mirrors in Kirkpatrick-Baez geometry at a distance of 1.475 and 1.000 m from the sample corresponding to geometrical source demagnification factors of 60 and 40 in horizontal (h) and vertical (v) directions, respectively. The mirror surfaces were optimized based on in-situ wave-front analysis and the highest quality regions of both mirrors were selected intercepting a 0.80(h) * 0.25(v) mm^2 cross-section of the incoming beam. These optimized settings provided a focal spot with an approximately Gaussian profile, a size of 5(h) x 4(v) μm^2 FWHM, and a total flux of $1.2*10^{12}$ photons/sec at the sample position. Background scattering was reduced by an in-vacuum high-precision slits system at 250 mm from sample position and by in-air capillary scatter guard 5 mm from the sample position.

All data were collected using an MD3 microdiffractometer (Cipriani et al. 2012) with a downward pointing vertically mounted omega-axis, designed by EMBL and built by ARINAX (Moirans, France).

The approximately flat surface of the cryo-loop was oriented parallel to the spindle-axis and normal to the X-ray beam using the mini-Kappa goniometer head MK3 (Cipriani et al. 2007, Brockhauser et al. 2013) mounted on the MD3. In this orientation it was possible to detect CatB crystals in the MD3 on-axis video microscope (OAV) (Cipriani & Castagna 2005) at highest magnification (0.143 µm/pixel) and center them to the beam. Preliminary diffraction images (Fig. S2, supporting information) were taken after centering the crystal to the calibrated position of the maximum beam position. Short rotation series (2-10°) were collected on a number of crystals with varying exposure conditions, including fine (oscillation range 0.1°, exposure times >40 ms) or coarse (oscillation range 0.5-1.0 degrees, exposure times 0.1 – 1 sec) slicing. XDS (Kabsch 2010) was used to index and integrate the diffraction images. The processing was successful for several data sets, but failed systematically on images or fine-sliced series collected with a total exposure time of less than 0.5 seconds, as well as on images acquired after the crystal was already exposed for 1 second or longer. Because only small and/or imprecisely measured wedges of data could be integrated on each sample, the attempts to quantify the crystal life time on the basis of these preliminary data were unsuccessful. Visual inspection of the images indicated that high resolution reflections started fading out already after 0.5 sec exposure.

The calculated absorbed dose rate was estimated using the program RADDOSE (Paithankar & Garman 2010), under the assumption of homogeneous crystal illumination with a photon flux density of 5.3×10^{10} ph/s/µm^2 in the maximum of the incident beam profile.

For the helical scans along the vertical direction, the capability of the MD3 to perform synchronized movements of the motors driving Ω axis and vertical goniometer alignment axis was applied. Once a line-scan was finished, the sample holder was moved by 5 µm in the horizontal direction to start the next line scan.

Our implementation of a serial data collection protocol utilized a subset of a more general implementation of continuous helical scans along a vector between two arbitrary points in 3D space developed for data collection on needle-shaped crystals (Cipriani, personal communication). The combined rotation-translations scan as implemented on the MD3 ensure that the deviations from an ideal trajectory are smaller than 0.3 µm and 0.001°, and the amplitude of erratic translational motion associated with rotation is smaller than 0.1 µm with an MK3 mini-kappa goniostat mounted. Thus, negative influences of mechanical or motion control imperfections on the TbCatB data, e.g. due to variations in the X-ray dose condition across the scanned area, are likely to be insignificant.

The variation in the total X-ray dose received by the sample was estimated taking into account a Gaussian beam profile to be between 60 MGy (for a point hit at the maximum of the incident beam

profile) and 50 MGy (for a point passed at 2.5 µm away from the maximum). Dose calculations did not account for photoelectron escape effects.

A3. Data processing

All collected diffraction images in CBF format were converted to HDF5 format for further analysis using CrystFEL (White et al. 2012). The CrystFEL software suite is, unlike any other conventional crystallographic data processing software, optimized to index single crystal diffraction images in random orientation, without collecting a continuous rotation series of one particular crystal. After extensive optimization of peak detection thresholds as well as experimental geometry, CrystFEL was used for the identification of indexable diffraction images. The total number of diffraction images for the final dataset was 28,800, of which 2,233 were identified as indexable diffraction patterns.

In the next step, diffraction images from line-scans that were indexed and adjacent to each other were grouped together. 595 of such groups containing between two and ten frames were identified, including 1734 frames in total (for a histogram, see figure S2). The groups of consecutive frames were further treated as regular rotation data in XDS. Spot search, indexing and integration were set up with default settings apart from the acceptance criteria for weak spots (allowing >= 2 pixel per spot and intensities => 2 sigma per pixel). The criterion of successful processing on the basis of the fraction of indexed spots was kept rather strict (> 50 %). Using an automated script to run XDS on all groups of consecutive frame resulted in successful processing of 130 groups corresponding to a total of 557 frames. The resulting 130 partial data sets were scaled together using XSCALE (Kabsch 2010) in two iterations whereby in the second iteration 10 partial data set showing overall correlation coefficients <0.70 with respect to the merged data in the first iteration where rejected. Finally, 109,661 reflection intensities in the resolution range 87-3.0 Å measured on a total of 426 diffraction images organized in 120 groups (the number of reflections per group varied between 350 and 2215) were merged. The fact that only a small fraction of frames that was successfully indexed by CrystFEL (426 out of 1734 corresponding to ca. 25 %) was included in the final data set is mostly due to the strict criteria applied in XDS restricting the accepted frames to the ones with strong diffraction signals. Attempts to relax the indexing criteria and to include weaker data into the scaling process did not lead to any significant changes in data statistics (as judged by the CC* criteria, data not shown). We anticipate that improvements in the data analysis procedure in particular with respect to the inclusion data from weak but accurately integrated diffraction images could lead to improved data statistics.

It should be noted that in the absence of geometrical reference between the diffracting volume and the incident beam profile, as in our experiment, it is impossible to assign a defined radiation dose to either individual frames or to the groups of frames. We believe that the high dose contributions >

30 MGy are negligible, due to the "global radiation damage" effects. Typically in MX, the diffraction power at 3.0 Å resolution reduces by a factor of 5 after exposure to 30 MGy (Kmetko et al. 2006, Holton 2009, Bourenkov & Popov 2010). Reflections affected by this loss in diffraction power will be filtered out during data reduction or at least be down-weighted during the data scaling and merging. In fact, attempts to reduce the high-dose contribution by deliberately truncating the last frames from each group did not lead to any improvement in data statistics (not shown). However, we cannot exclude that the optimal exposure time for the samples used would have been shorter than 1 second/frame.

A4. Data quality evaluation

The quality and internal consistency of the data was judged on the basis of standard $<I/\sigma I>$ statistics and on the basis of the CC* criteria recently advocated as a single statistically valid guide for deciding which data are useful (Karplus & Diedrichs 2012, Evans 2012, Evans & Murshudov 2013). CC* estimates the correlation coefficient to the set of unknown "ideal" data on the basis of the sample statistics derived from two random half-datasets, for TbCatB data the CC* calculated in resolution shells (Table S1, supporting information) indicated the presence of statistically significant data to a resolution of 3.0 Å and below.

Rmerge/Rmeas values in Table S1 are calculated on the basis of all data included in scaling, i.e. no individual reflections were rejected; if inconsistent data were detected, groups of entire diffraction images were rejected as a whole. The high Rmerge/Rmeas values observed here are mostly due to the presence of a substantial number of redundant low-precision measurements arising from the smallest crystals, measurements made in the tails of the beam or after a crystal received high radiation dose. It should be noted that the standard R-factor analysis fundamentally assumes similar magnitudes of intensity measurements errors for equivalent reflections (Diederichs & Karplus, 1997); this is not the case for our data. In fact, merging R-factors do not appear to be a useful criterion for judging the data quality in a serial experiment on samples delivering only weak but still variable signals.

The standard $<I/\sigma I>$ statistics of the TbCatB data presented here (Table 1) can be compared to that of the cubic lipid phase GPCR data obtained by merging similar numbers (30 to 80) of partial data sets, each measured at a very high radiation dose (Hanson et al. 2012, Hollenstein et al. 2013). In the high resolution shells $<I/\sigma I>$ of TbCatB and GPCR data are similar, while for low resolution shells $<I/\sigma I>$ are lower for TbCatB than for the GPCR data. This is a consequence of the fact that TbCatB crystal volumes (5-20 μm^3) are typically two orders of magnitude smaller than those of the GPCR crystals (500-2000 μm^3), resulting in proportionally lower scattering power in all resolution shells. Notably, the slope of the average reflection intensity vs. resolution, as characterized by average temperature

factors is much steeper for GCPRs ($<B_{All}>\approx 70$ Å^2) than for TbCatB ($<B_{All}>\approx 41$ Å^2). The crystal volume (<I> intercept) and average B (<I> slope) effects in combination lead to a noticeably lower signal-to-noise ratio at low- to medium- resolution, but similar limiting resolution. In terms of CC* criterion, this effect is reflected by unusually low values CC* of 0.98-0.99 in low resolution shells for TbCatB (*vs* CC*> 0.999 for a typical well diffracting single crystal). Finally, the structure solution process, refinement and comparison to high-resolution SFX structure were used to validate the data significance and to estimate the resolution.

A5. Structure Determination

The serial synchrotron diffraction data were phased by molecular replacement using PHASER (McCoy et al. 2007) and the coordinates of the mature active form of TbCatB as a search model (Protein Data Bank ID, 3MOR), which exhibits an amino acid sequence identity of 100 % to the *T. brucei* procathepsin B determined in this study, except the missing propeptide sequence. During different stages of model building and refinement using COOT (Emsley & Cowtan 2004) and REFMAC5 (Murshudov et al. 1997), respectively, 62 propeptide residues, and 5 carbohydrate residues were placed in a difference electron density map. Water and glycerol molecules as well as ions were indicated to be present by difference electron density peaks but could not be refined with confidence and therefore were not included in the model. The synchrotron structure of the TbCatB-propeptide complex was refined at a resolution of 3.0 Å to an *R*-factor of 22.3 % (R_{free} = 26.4 %). Refinement statistics are summarized in **Table 1**.

A6. Structure analysis and illustrations

Superposition and RMSD calculations have been performed using the program SUPERPOSE of the CCP4i software suite (Maiti et al. 2004). All illustrations were prepared using PyMol v1.3 (DeLano Scientific; http://www.pymol.org).

Table S1 Data statistics in resolution shells.

Resolution (Å)	# reflections		Completeness (%)	R_{merge}[a]	R_{meas}	<I//σI>	CC^{*b}
	Observed	Unique					
9.48	3067	330	97.6	0.301	0.317	8.0	0.988
6.70	6204	550	100.0	0.349	0.366	7.0	0.988
5.47	8464	688	100.0	0.472	0.492	5.1	0.974
4.74	9914	807	99.9	0.484	0.506	5.23	0.968
4.24	11324	890	99.9	0.489	0.510	5.77	0.968
3.87	12482	973	100.0	0.615	0.640	4.40	0.973
3.58	13834	1072	100.0	0.826	0.860	3.23	0.952
3.35	13578	1133	99.7	1.103	1.152	2.27	0.911
3.16	15008	1191	99.9	1.620	1.688	1.59	0.858
3.00	15706	1252	99.9	2.690	2.806	1.01	0.787

[a] $R_{merge} = \frac{\sum_{hkl}\sum_{i=1}^{n} | I_i\,(hkl) - \bar{I}(hkl)|}{\sum_{hkl}\sum_{i=1}^{n} I_i\,(hkl)}$ $CC^* = \sqrt{\frac{2\,CC_{1/2}}{1+\,CC_{1/2}}}$

[b] $CC_{1/2}$ as output by XSCALE.

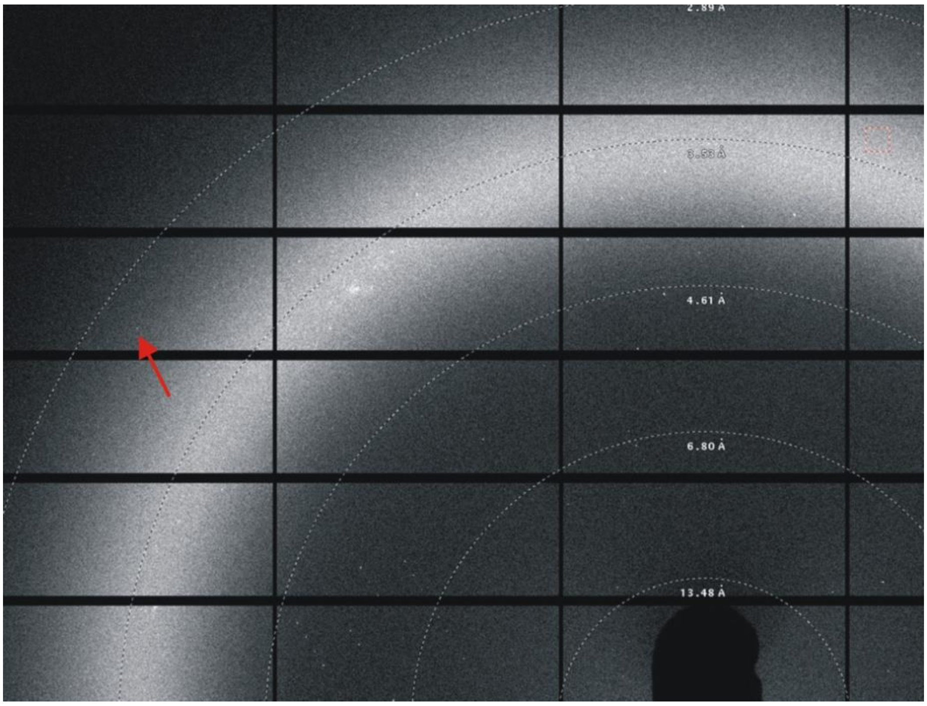

Figure S1 Single crystal diffraction pattern of *in vivo* crystallized TbCatB recorded within an oscillation range of 1° during an exposure time of 2 s. Bragg diffraction spots are visible up to a maximum resolution of approximately 3 Å. The red arrow exemplarily marks a high resolution diffraction spot.

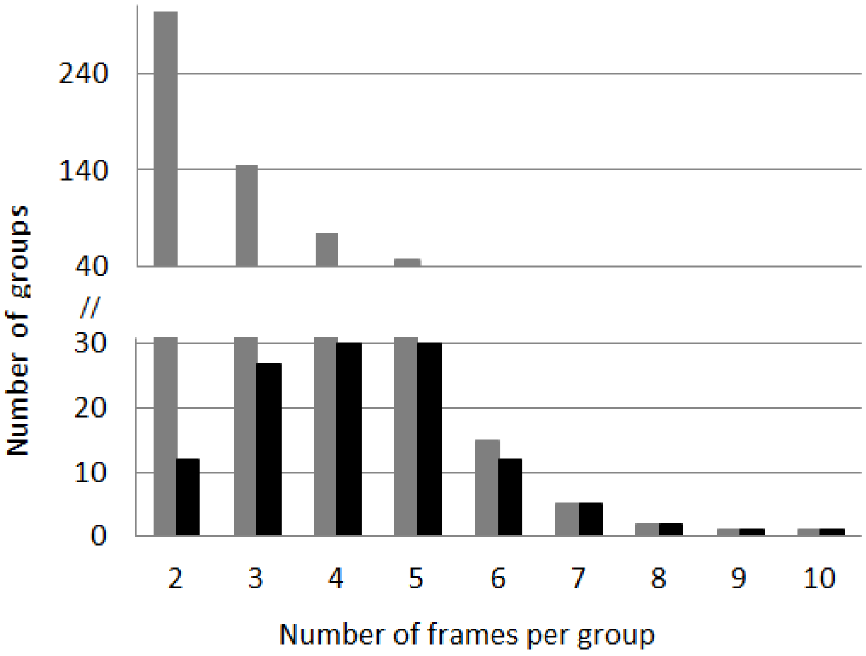

Figure S2 Size distribution in the groups of adjacent indexed frames. Gray bars indicate the number of groups indexed by CRYSTFEL. Black bars indicate the number of groups included in the final data set.

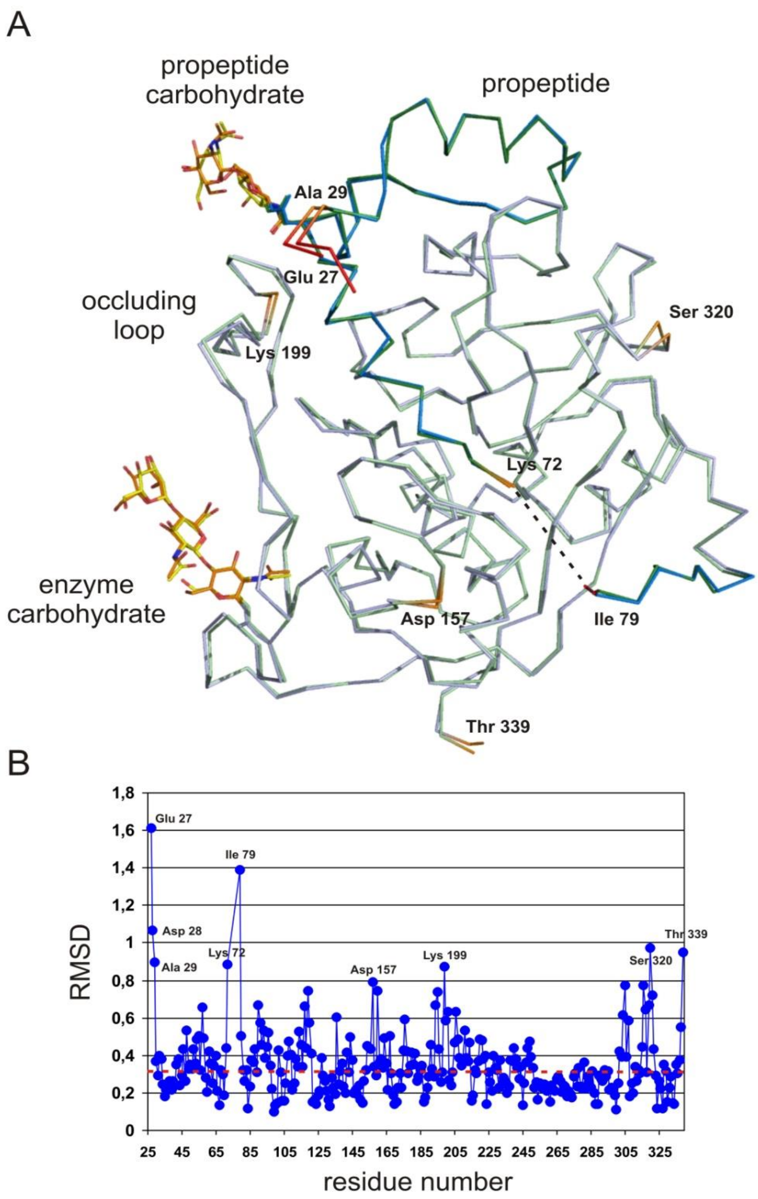

Figure S3 (a) Comparison of the TbCatB structures determined by serial crystallography approaches with synchrotron and FEL radiation. The Cα plot of the TbCatB-propeptide complex obtained by application of the serial synchrotron diffraction method at 100 K (3.0 Å resolution, green/yellow) is superimposed on that obtained by SFX techniques at room temperature at an FEL source (refined to 3.0 Å resolution, blue/orange, Protein Data Bank ID, 4HWY)[31]. Carbohydrate chains displayed in stick representation are colored in yellow and orange, respectively. (b) Significant structural deviations of more than 0.8 Å (orange) and 1.0 Å (red) are restricted to residues located in loop structures (Asp157, Lys199, and Ser322), at the N- and C-terminus (Ala27, Asp28, Glu29, and Thr339) as well as at positions flanking the disordered part of the propeptide (Lys72 and Ile79), consisntenly characterized by increased flexibility. The average RMS deviation between the enzyme structures of 0.35 Å for 307 equivalent Cα atoms is indicated in the diagram (red line).

Supplementary references

Bourenkov, G.P. & Popov, A.N. (2010). *Acta Crystallogr. D Biol. Crystallogr*. **66**, 409-419.

Brockhauser, S., Ravelli, R.B.G., McCarthy, A.A. (2013). *Acta Crystallogr. D Biol Crystallogr.* **69**, 1241-1251.

Cipriani, F., Felisaz, F., Lavault, B., Brockhauser, S., Ravelli, R., Launer, L., Leonard, G., Renier, M. (2007). *AIP Conf. Proc.* **879**, 1928-1931.

Cipriani, F., Castagna, J.C. US patent 6927399 B2. Aug. 9 2005.

Cipriani, F., Feisaz, F., Landret, C., Gobbo, A., Oss, D. & Bourenkov, G. (2012). 11th International Conference on Synchrotron Radiation Instrumentation, SRI.

Diederichs, K. & Karplus, P.A. (1997). *Nat. Struct. Mol. Biol.* **4**, 269-275.

Emsley, P. & Cowtan, K. (2004). *Acta Crystallogr. D Biol. Crystallogr.* **60**, 2126-2132.

Hanson, M.A., Roth, C.B., Jo, E., Griffith, M.T., Scott, F.L., Reinhart, G., Desale, H., Clemons, B., Cahalan, S.M., Schuerer, S.C., Sanna, M.G., Han, G.W., Kuhn, P., Rosen, H., Stevens, R.C. (2012) *et al. Science* **335**, 851-855.

Hollenstein, K., Kean, J., Bortolato, A., Cheng, R.K., Doré, A.S., Jazayeri, A., Cooke, R.M., Weir, M., Marshall, F.H. (2013). *Nature* **499**, 438-443.

Holton, J.M. (2009). *J. Synchr. Radiat*. **16**, 133-142.

Kmetko, J., Husseini, N.S., Naides, M., Kalinin, Y., Thorne, R.E. (2006). *Acta Crystallogr. D Biol. Crystallogr.* **62**, 1030-1038.

Maiti, R., Van Domselaar, G.H., Zhang, H., Wishart, D.S. (2004). *Nucleic Acids Res.* **32**, 590-594.

Murshudov, G. N., Vagin, A., Dodson, E. J. (1997). *Acta Crystallogr. D Biol. Crystallogr.* **53**, 240-255.
